# Ehrlich Tumor Induces TRPV1-Dependent Evoked and Non-Evoked Pain-like Behavior in Mice

**DOI:** 10.3390/brainsci12091247

**Published:** 2022-09-15

**Authors:** Mariana M. Bertozzi, Telma Saraiva-Santos, Tiago H. Zaninelli, Felipe A. Pinho-Ribeiro, Victor Fattori, Larissa Staurengo-Ferrari, Camila R. Ferraz, Talita P. Domiciano, Cassia Calixto-Campos, Sergio M. Borghi, Ana C. Zarpelon, Thiago M. Cunha, Rubia Casagrande, Waldiceu A. Verri

**Affiliations:** 1 Laboratory of Pain, Inflammation, Neuropathy, and Cancer, Department of Pathology, Center of Biological Sciences, Londrina State University, Londrina 86057-970, PR, Brazil; 2 Division of Dermatology, Department of Medicine, Washington University School of Medicine, St. Louis, MO 63110, USA; 3 Center for Research in Health Sciences, University of Northern Londrina, Londrina 86041-120, PR, Brazil; 4 Department of Pharmacology, Ribeirão Preto Medical School, University of São Paulo, Avenida Bandeirantes, Ribeirão Preto 14049-900, SP, Brazil; 5 Department of Pharmaceutical Sciences, Center of Health Science, Londrina State University, Londrina 86038-440, PR, Brazil

**Keywords:** AMG9810, calcium imaging, cancer pain, dorsal root ganglia, Ehrlich tumor, mechanical hyperalgesia, nociception, pain, thermal hyperalgesia, TRPV1

## Abstract

We standardized a model by injecting Ehrlich tumor cells into the paw to evaluate cancer pain mechanisms and pharmacological treatments. Opioid treatment, but not cyclooxygenase inhibitor or tricyclic antidepressant treatments reduces Ehrlich tumor pain. To best use this model for drug screening it is essential to understand its pathophysiological mechanisms. Herein, we investigated the contribution of the transient receptor potential cation channel subfamily V member 1 (TRPV1) in the Ehrlich tumor-induced pain model. Dorsal root ganglia (DRG) neurons from the Ehrlich tumor mice presented higher activity (calcium levels using fluo-4 fluorescent probe) and an increased response to capsaicin (TRPV1 agonist) than the saline-injected animals (*p* < 0.05). We also observed diminished mechanical (electronic von Frey) and thermal (hot plate) hyperalgesia, paw flinching, and normalization of weight distribution imbalance in TRPV1 deficient mice (*p* < 0.05). On the other hand, TRPV1 deficiency did not alter paw volume or weight, indicating no significant alteration in tumor growth. Intrathecal injection of AMG9810 (TRPV1 antagonist) reduced ongoing Ehrlich tumor-triggered mechanical and thermal hyperalgesia (*p* < 0.05). Therefore, the contribution of TRPV1 to Ehrlich tumor pain behavior was revealed by genetic and pharmacological approaches, thus, supporting the use of this model to investigate TRPV1-targeting therapies for the treatment of cancer pain.

## 1. Introduction

In 1905, Paul Ehrlich described a mammary adenocarcinoma that was found to develop spontaneously in mice [1]. Ehrlich tumor cells are maintained by intraperitoneal (i.p.) passages in an ascitic form, which is used for pharmacological screening and pathophysiological investigation [2,3]. The Ehrlich tumor cells can also develop as a solid tumor when injected subcutaneously [4]. We developed an Ehrlich tumor pain model by injecting these cells subcutaneously into the plantar side of the hind paw of mice [5]. In this model, we observed that Ehrlich tumor cells induce pain-like behavior that was amenable to treatment with the opioid morphine. On the other hand, the cyclooxygenase inhibitor indomethacin and the tricyclic antidepressant amitriptyline were ineffective in altering pain-like behavior [5]. Breast cancer patients report pain in other phases in addition to the metastasis phase. In fact, 1/3 of those patients report spontaneous pain in the lump and when pressure is applied during examination [6]. Preoperative breast pain correlates with phantom breast pain syndrome, and adequate analgesia before mastectomy can diminish phantom breast pain syndrome [7]. Our interpretation is that the model developed by our group resembles preoperative breast cancer pain in the lump and could be useful to screen and understand the pathophysiological mechanisms of such conditions, which ultimately can lead to novel treatments [5].

Transient receptor potential cation channel subfamily V member 1 (TRPV1) is important in pain processing. Indeed, most nociceptive C fibers express TRPV1 and serve to sense peripheral alterations and transmit these nociceptive inputs to the spinal cord. TRPV1 senses many types of alterations: for instance, TRPV1 senses noxious heat (>42 °C), extreme acidic or basic pH, lipids such as lysophosphatidic acid, chemicals with pungent characteristics (e.g., chili pepper compound capsaicin), and toxins (e.g., vanillotoxins) [8,9]. Supporting the significance of TRPV1 in cancer pain, targeting TRPV1 with genetic and pharmacological approaches reduces pain in bone cancer [10,11], squamous cell carcinoma [12], and pancreatic cancer [13]. Although each cancer type has its own peculiarities, identifying drug-targetable pathophysiological mechanisms is essential to developing novel analgesic therapies. Therefore, in this study, we evaluated the contribution of TRPV1 to Ehrlich tumor pain.

## 2. Material and Methods

### 2.1. Animals

Male C57BL/6 and TRPV1 deficient (TRPV1^-/-^, C57BL/6 background) mice for this study were from Londrina State University (Paraná, Brazil) and Ribeirão Preto Medical School, University of Sao Paulo (São Paulo, Brazil), respectively. All mice weighed 22 ± 1 g, 8–9 weeks-old, and were housed in standard clear plastic cages, with a standard light/dark cycle of 12:12 h, and had food and water ad libitum. The temperature was maintained at 21 ± 1 °C and behavioral experiments occurred in the light cycle between 9 a.m. and 5 p.m. To achieve equal sample size at all time points, mice were separated by block randomization. The ARRIVE 2.0 (Lilley et al., 2020) and International Association for Study of Pain (IASP) guidelines were followed and the study was designed to minimize the number of animals and their suffering as much as possible. Prior approval by the Londrina State University Ethics Committee on Animal Research and Welfare was obtained (process number 14543.2013.03). Inhalation of isoflurane (5% oxygen using a precision vaporizer) was applied to euthanize animals, and then decapitation was performed as a confirmatory method. This study used a total of 144 C57BL/6 mice and 20 TRPV1^-/-^ C57BL/6 mice.

### 2.2. Experimental Procedures

Ehrlich cells (1 × 10^6^) were suspended in sterile saline (25 µL) and administrated by subcutaneous plantar injection (intraplantar [i.pl.]). On the 12th day, mice were euthanized with isoflurane 5%, and the DRG from L4-L6 were collected, processed to isolate neurons, and cultured overnight. Fluo-4-AM probe loading into DRG neurons allowed detecting by fluorescence the kinetics of intracellular calcium as a surrogate measure of neuronal activity. Basal levels of calcium and upon capsaicin stimulation were quantitated by calcium imaging using a confocal microscope. In another set of experiments, TRPV1^-/-^ and C57BL/6 mice received an i.pl. injection of Ehrlich tumor cells. Mechanical hyperalgesia (electronic aesthesiometer, an apparatus similar to von Frey filaments, but in an electronic version), thermal hyperalgesia (hot plate apparatus), paw thickness (caliper), and the distribution of weight between the hind paws (static weight bearing apparatus) were determined at the basal time point and every two days until the 12th day. After these measurements were taken, the animals were euthanized, and the paws were collected and weighed. To investigate the effects of TRPV1 pharmacological inhibition, Ehrlich tumor cells (i.pl.) were administered to C57BL/6 mice and mechanical and thermal hyperalgesia were assessed at baseline (before injection) and on the 8th day post Ehrlich tumor injection. On the 8th day post Ehrlich tumor administration, mice were treated with AMG9810 (intrathecally, 100 nmols) or vehicle (2% DMSO in saline), and mechanical and thermal hyperalgesia were evaluated at the indicated time points. The experimental conditions, measurement time points, Ehrlich tumor cell load, and AMG9810 dose of treatment were determined in prior studies [5,14,15].

### 2.3. Calcium Imaging

We followed a prior description of calcium imaging in DRG neurons [15]. On the 12th day after stimulus, C57BL/6 Ehrlich- or saline-stimulated DRG neurons were dissected into Neurobasal-A medium (Life Technologies, Thermo Fisher Scientific, Waltham, MA, USA) following a dissociation step using enzymes (collagenase A [1 mg/mL]/dispase II [2.4 U/mL] RocheApplied Sciences, Indianapolis, IN, USA) diluted in HEPES-buffered saline medium (MilliporeSigma, Burlington, MA, USA) and incubated at 37 °C for 15 min. Following the dissociation step the cells were centrifuged for 5 min at 300× *g*. The cells were then resuspended in 800 µL of DMEM supplemented with 10% FBS and DNase I. Glass Pasteur pipettes of progressively decreasing sizes were sequentially used to dissociate DRG neurons. This step was followed by centrifugation of 30 min at 1200 rpm, applying a 15% BSA gradient. The resulting pellet consisted of DRG neurons, which were resuspended, counted and plated in Neurobasal-A medium containing nerve growth factor (NGF, 50 ng/mL, Life Technologies, Thermo Fisher Scientific, Waltham, MA, USA). We used a total of 5000 DRG neurons per dish, which were plated overnight on culture dishes with a laminin coating. On the following day, DRG cells were loaded with 1.2 µM of Fluo-4AM in Neurobasal-A medium and incubated at 37 °C for 30 min. Neurons were washed with Hanks’ Balanced Salt Solution three times (HBSS calcium, magnesium, no phenol red, GIBCO, #14025092, Waltham, MA, USA). Fluorescence imaging was carried out with a TCS SP8 Confocal Microscope (Leica Microsystems, Mannheim, Germany). To assess TRPV1 activation, DRG plates were recorded for a total time of 7 min, which was divided into: baseline values/initial reading (2 min), TRPV1 stimulation with its agonist capsaicin (100 nM in HBSS, MilliporeSigma, Burlington, MA, USA) for up to 4 min, and then KCl solution (final concentration was 40 mM) for the remainder of the recording time. The TCS SP8 confocal microscope used the LAS X Software (Leica Microsystems, Mannheim, Germany) [16,17,18,19,20], which was applied to analyze the mean fluorescence intensity representing calcium influx.

### 2.4. Mechanical Hyperalgesia 

The von Frey’s filaments evolved into an electronic apparatus that can be used with interchangeable probes while maintaining a fixed probe and area of contact during a set of experiments. This testing is described to measure mechanical hyperalgesia when there is a basal response and alteration following stimulation and treatments [14]. Similarly to other behavioral testing, animals must be placed in a quiet room and gently placed in cages (12 × 10 × 17 cm) that allow access to the paws from below through wire grid floors. At least 45 min of habituation to the testing room must be carried out on the day of the experiment; however, prior sessions of habituation lasting 60 min were also conducted on the four consecutive days leading up to the experiment. Following habituation on the day of the experiment, the handheld force transducer (electronic aesthesiometer, Insight instruments, Ribeirao Preto, SP, Brazil) [14] was used to apply a perpendicular stimulation on the hind paw. A 0.5 mm^2^ polypropylene tip was placed in the handheld force transducer to allow the stimulation of a constant area. The endpoint was characterized by the removal and flinching of both hind paws. The apparatus records the measured value automatically. Measurements were performed in basal conditions as well as after stimulation and treatment. The mean mechanical withdrawal threshold of three values (in grams) is shown in figures for each time point during the 12 days. An ongoing TRPV1-blocking evaluation was performed for the pharmacological treatment; the mechanical threshold was determined before, 1 h, 3 h, 5 h, and 7 h after intrathecal treatment with AMG8910 (100 nmols, 10 μL, Cayman Chemical, Ann Arbor, MI, USA) [21]. The investigators were blinded to the treatment.

### 2.5. Thermal Hyperalgesia 

A hot plate apparatus was set at 52 ± 1 °C to assess heat thermal hyperalgesia [22]. The experimenter was trained to identify hind paw removal from the plate and immediate flinching or licking behaviors, which were considered the characteristic endpoint. The response latency was recorded before Ehrlich tumor cells injection (i.pl.) and every two days up to the 12th day. Heat thermal hyperalgesia was also determined on the 8th day, before, 1 h, 3 h, 5 h, and 7 h after intrathecal treatment with AMG8910 (100 nmols, 10 µL) [21]. Considering tissue damage can occur depending on the time of exposure, we used a cut-off of 20 s [22]. The investigators were blinded to the treatment.

### 2.6. Tumor Growth: Paw Thickness and Paw Weight 

Paw thickness was determined using an analog caliper thickness gauge (Mitutoyo 7301 A, Mitutoyo Corporation, Kanagawa, Japan) [19,20] before and after Ehrlich tumor cells injection at the indicated time points (every two days). Paw thickness/tumor growth was quantitated in mm [23]. At the end of the 12 days, the weight of the contra and ipsilateral paws were compared, and the delta paw weight was evaluated as a measure of tumor growth. Paw weight was presented as Δipsi-contra lateral grams (g) [24].

### 2.7. Static Weight Bearing (SWB)

Disability in weight distribution in the mice’s paws was evaluated before and after induction of cancer-related pain for 12 days. This analysis was made using an apparatus (Model BIO-SWB-TOUCH-M, Bioseb, France) [25,26] specifically developed to assess SWB. Similarly to the other tests, the mice were handled gently in a quiet temperature-controlled room and underwent four days of habituation. The apparatus consists of an acrylic chamber with two pressure plates in which the hind paws are placed. Mice were placed into an acrylic chamber allowing the mice to adjust their spontaneous positioning. The apparatus quantitates the weight value distributed to each sensor [27]. We used the mean of three measurements at 0 (baseline value) and after Ehrlich Tumor Cells injection (1 × 10^6^ cells, 25 µL in sterile saline, i.pl.) to calculate the Right/Left paw ratio. Measurements were performed every two days after induction and until day 12. The investigators were blinded to the groups.

### 2.8. Statistical Analysis

Results are presented as the mean ± SEM for parametric data and median + range for non-parametric data. To this end, we used Shapiro–Wilk normality test and Brown-Forsythe homogeneity test. In in vitro experiments, a n of 2 plates of DRG neuron cultures were used in each group per experiment, and each plate was a pool of DRGs of 10 animals. These in vitro experiments were performed twice and the results are shown as the mean of each experiment, thus each dot is the mean of a separate in vitro experiment. For in vivo experiments, a n of 10 mice was used in each group for the effect of TRPV1 deficiency, and a n of 8 mice was used in each group for the experiments with AMG9810 intrathecal treatment. Data were analyzed using the software GraphPad Prism version 9.2 (La Jolla, CA, USA). Data from experiments of multiple time points and containing at least three groups for comparison (mechanical, thermal hyperalgesia, paw thickness, paw weight, and static weight bearing) were analyzed using Kruskal–Wallis followed by Dunn post-test or two-way repeated measure analysis of variance (ANOVA) followed by Tukey’s post hoc, depending on whether results were non-parametric or parametric, respectively. For parametric data from experiments with a single time point, one-way ANOVA, followed by Tukey’s post hoc, was used. Statistical differences were considered significant when *p* < 0.05.

## 3. Results

### 3.1. Ehrlich Tumor Cells Induce DRG Neuronal Activation and Enhance Capsaicin Response 

**Figure 1:**
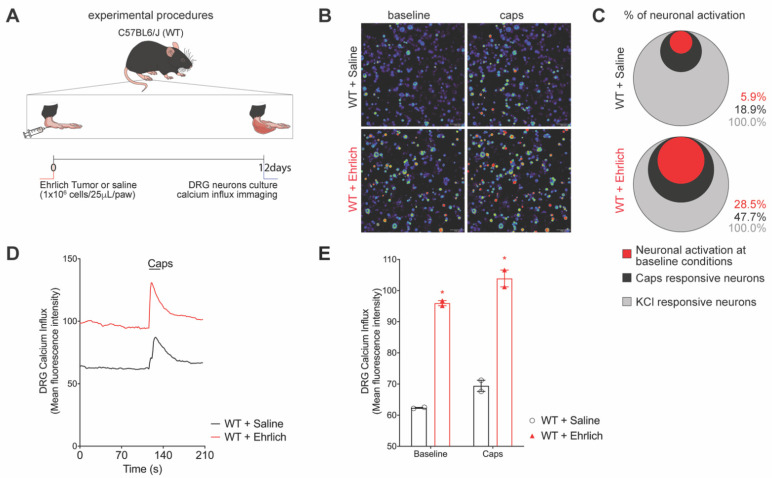
DRG neurons from Ehrlich tumor-stimulated mice presented with high basal activation and enhanced response to capsaicin, a TRPV1 agonist. (**A**) Experimental design scheme. C57Bl/6 mice received an intraplantar injection of Ehrlich tumor cells (10^6^, 25 µL paw) or saline in the control group and 12 days after the DRGs (L4–L6) were collected and cultured. (**B**) Representative fields of baseline fluorescence of DRG neurons dissected from saline and Ehrlich tumor cells-stimulated mice and representative fields after capsaicin plate stimulation (1 μM per plate). (**C**) Venn Diagram comparing the neurons population with increased baseline activation that had responded to capsaicin stimulation. (**D**) Tracers of mean fluorescence intensity of calcium influx of the baseline (0-s mark) and following capsaicin stimulation (120 s mark, TRPV1 agonist). The black line refers to the Saline-stimulated DRGs, and the red line refers to the Ehrlich tumor cells-stimulated DRGs. (**E**) Quantification of mean fluorescence intensity of calcium influx of baseline (0-s mark) and following the stimulus with capsaicin (120-s mark). *n* = 2 DRG culture plates per group per experiment. Each plate is a neuronal culture pooled from 10 mice. Two independent experiments were performed, and plotted data are the mean of both experiments. Results are expressed as median + range. (* *p* < 0.05 vs. saline). Statistical information can be found at Table S1.

We first addressed if Ehrlich tumor cells administration in vivo would induce the activation of DRG neurons. Peak pain is achieved on the 8th day post-tumor injection and this pattern is maintained up to the 12th day of the model [5]. On the 12th day, the right side of the DRGs from L4 to L5 were dissected, digested, and loaded with fluo-4AM for calcium imaging as a surrogate for neuronal activation. Plantar Ehrlich tumor cells increased the basal levels of intracellular calcium and the capsaicin-triggered influx (Figure 1A,B). DRG neurons of mice bearing plantar Ehrlich tumor cells presented a higher percentage of activated neurons at baseline conditions with augmented capsaicin responsiveness (Figure 1C), which can also be observed by calcium fluorescence intensity representative tracers (Figure 1D) and quantitation (Figure 1E). Table S1 presents the statistical analyses in detail.

### 3.2. TRPV1 Deficiency Reduces Ehrlich Tumor Cells Triggered Pain-like Behavior without Changing Parameters of Tumor Growth

**Figure 2:**
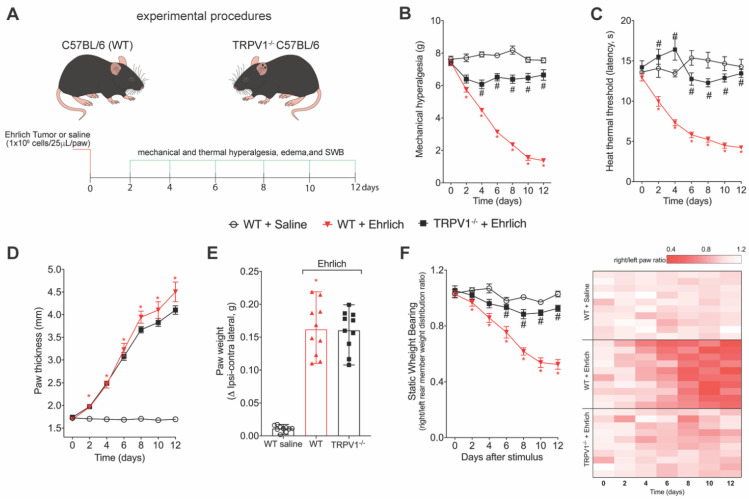
TRPV1 deficiency reduces Ehrlich tumor cells pain-like behavior without changing tumor growth parameters. (**A**) Experimental design. Male WT (Wild type) C57BL/6 and TRPV1 (transient receptor potential cation channel subfamily V member 1) deficient (^-/-^) (C57BL/6 background) mice were stimulated with intraplantar Ehrlich tumor cells at the load of 1 × 10^6^ cells or saline on day zero. Behavior measures were performed after injecting the stimuli to set up the baseline. After the induction of the model, the behavior parameters were performed every two days up to the 12th day. (**B**,**C**) Mechanical and thermal hyperalgesia were evaluated using an electronic version of von Frey filaments and hot plate apparatus, respectively. (**D**) Paw thickness was evaluated using analog calipers, expressing the results in mm. In addition, the ipsilateral and contralateral paws were weighed, and the delta in paw weight was evaluated as a measure of tumor growth (**E**). The depletion of TRPV1 in mice did not alter the pattern of tumor growth. Results are presented as the mean ± SEM of mechanical threshold in grams (electronic von Frey) or heat thermal threshold latency in seconds (hot plate). *n* = 10 mice per group (* *p* < 0.05 vs. saline group; # *p* < 0.05 vs. Ehrlich tumor cells group. (**E**) Results are expressed as median + range of paw weight ratio. *n* = 10 mice per group (* *p* < 0.05 vs. saline group). (**F**) SWB was used as a non-reflexive method of pain measurement. The heat map shows Right/Left rear paw ratio of each mouse. Results are presented as the weight ratio of Right/Left rear paw. *n* = 10 mice per group (* *p* < 0.05 vs. saline group; # *p* < 0.05 vs. Ehrlich tumor cells group). Statistical information can be found at Table S2.

We next evaluated the participation of TRPV1 in Ehrlich-induced pain. Ehrlich tumors induced a significant decrease in evoked nociceptive thresholds upon mechanical (Figure 2B) or thermal (Figure 2C) stimulation. Ehrlich tumors also caused an imbalance in weight bearing distribution towards the opposite paw (Figure 2F). TRPV1^-/-^ mice did not develop mechanical (Figure 2B) or thermal hyperalgesia (Figure 2C), or imbalance in weight bearing distribution (Figure 2F). Importantly, TRPV1 deficiency did not interfere in Ehrlich tumor growth, as demonstrated by paw thickness (Figure 2D) and paw weight (Figure 2E) data. Thus, TRPV1 deficiency reduced pain-like behavior without interfering with tumor growth. Table S2 presents the statistical analyses in detail. 

### 3.3. The TRPV1 Antagonist AMG9810 Reduces Ongoing Hyperalgesia Induced by Ehrlich Tumor Cells

**Figure 3:**
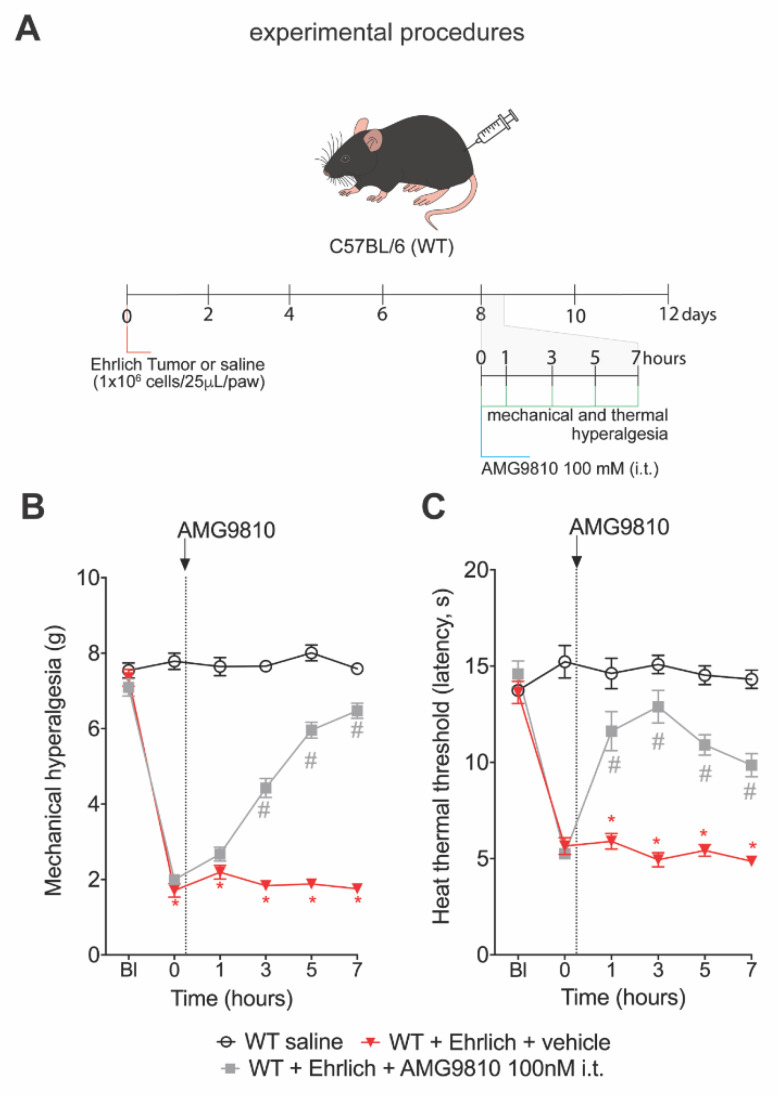
TRPV1 pharmacological blocking reduces Ehrlich tumor cells pain-like behavior. (**A**) Experimental design. Male WT (Wild type) C57BL/6 mice were stimulated with intraplantar Ehrlich tumor cells (10^6^ cells, 25 μL paw). Furthermore, on the 8th day, when peak pain-like behavior was achieved, AMG9810 (100 nM, 10 μL intrathecal, competitive antagonist of TRPV1) was administered, and animals were evaluated 1 h, 3 h, 5 h, and 7 h after intrathecal treatment. (**B**) Ehrlich tumors triggered ongoing mechanical hyperalgesia and (**C**) thermal hyperalgesia and were reduced by treatment with AMG9810 compared to the vehicle (2% dimethyl sulfoxide in saline)-treated group. Results are presented as the mean ± SEM of mechanical threshold in grams (von Frey) or heat thermal threshold latency in seconds (hot plate). *n* = 8 mice per group (* *p* < 0.05 vs. saline group; # *p* < 0.05 vs. Ehrlich tumor cells group. Statistical information can be found at Table S3.

We also addressed whether pharmacological inhibition of TRPV1 could reduce ongoing hyperalgesia triggered by Ehrlich tumors. Mice received Ehrlich tumor cells in the paw and at the 8th day received AMG9810 or its vehicle (2% DMSO plus 98% of saline) intrathecally, which delivers the drug to the spinal cord and DRGs. AMG9810 reduced the Ehrlich tumor cell-triggered mechanical (Figure 3B) and thermal hyperalgesia (Figure 3C). Thus, genetic deletion (Figure 2) and pharmacological inhibition (Figure 3) demonstrate TRPV1 involvement in pain-like behavior that occurs upon administration of Ehrlich tumor in mice. Table S3 presents the statistical analyses of Figure 3 in detail.

## 4. Discussion

The present work shows that Ehrlich tumor cells can induce the activation of DRG neurons and enhance neuronal activation upon capsaicin stimulation. Corroborating these data, interfering with TRPV1 using genetic and pharmacological approaches reduced Ehrlich tumor-triggered pain-like behavior without affecting tumor growth. To our knowledge, this is the first study demonstrating TRPV1 contribution to Ehrlich tumor-induced pathology. 

TRPV1 expression characterizes non-myelinated peptidergic neurons called C-fibers, which mainly function as nociceptors, although some of the C-fibers also contribute to pruritus [28,29,30,31]. These polymodal nociceptors are involved in a great variety of painful conditions ranging from inflammatory pain to neuropathic pain, complex regional pain syndrome type 1, and cancer pain [32,33]. For instance, in inflammatory pain, in addition to neurotransmission of nociceptive inputs, TRPV1^+^ neurons, through the release of neuropeptides, participate in generating neurogenic inflammation and communicate with immune cells, regulating their recruitment and function such as phagocytosis [18]. Thus, TRPV1^+^ neurons have a role in immune regulation during disease. Similarly, cancer pain also involves the interaction of cancer cells, immune cells, and neurons [34]. In a model of melanoma, conditional knockdown or ablation of nociceptive neurons led to enhanced tumor progression [35,36]. These data support that nociceptive neurons may have an endogenous protective role against cancer progression. However, TRPV1 deficiency did not alter paw thickness and paw weight. Although these are relatively gross measurements, any significant effect on tumor progression can be detected using such parameters [24,37,38]. Thus, it is likely that TRPV1^+^ neurons are not major players in shaping Ehrlich tumor growth.

This lack of contribution of TRPV1^+^ neurons to neuro-immune-cancer interactions supports the rationale that as TRPV1 deficiency reduces pain-like behavior, the major function of this TRP channel in the Ehrlich tumor model is neuronal activation and neurotransmission of nociceptive inputs. Evidence supports that tumor cells cause an acidic environment and TRPV1 senses the enhancement of protons in tissues, leading to neuronal activation and pain [39,40,41]. Ehrlich tumor cells also trigger the production of hyperalgesic cytokines [38] that sensitize nociceptor neurons through the increase in activity and expression of ion channels [33,42,43]. Therefore, the present results are consistent with the idea that Ehrlich tumor progression would enhance pain-like behavior over time, and TRPV1 inhibition would reduce pain without a significant role in tumor progression.

We used two approaches to target TRPV1. The TRPV1 deficiency was used to verify its contribution over time and offers the complete deletion in the host. The pharmacological inhibition using AMG9810 was used to verify whether TRPV1 inhibition would have an effect against ongoing pain, and to confirm whether the effect of TRPV1 deficiency is related to the targeted deletion or a mistargeted adaptation. Furthermore, with AMG9810, we could use the intrathecal treatment to isolate its effect in two anatomic regions, the DRG neurons and spinal cord, instead of a complete animal deletion considering evidence supporting peripheral immune cells such as macrophages also express TRPV1 [44,45,46]. These approaches indicate that in Ehrlich tumors, TRPV1 contributes to pain without affecting tumor progression; thus, therapies targeting TRPV1 could be useful. There are other options for interfering with TRPV1 activity. For instance, evidence supports that phosphorylation by PKC (protein kinase C) of the TRPV1 S801 contributes to ligand-induced activation of this channel but not to heat activation, as observed by knock-in alanine in TRPV1 S801 [47].

TRPV1 is straightforwardly related to thermal hyperalgesia and overt pain-like behavior such as paw flinching, which is a result of the activation of such channels [48,49,50,51]. However, inhibiting TRPV1 also results in reduced mechanical hyperalgesia [52,53,54,55]. This outcome might be related to the release of neuropeptides by TRPV1 neurons, which can activate immune cells such as macrophages [56]. In turn, macrophages would produce mediators that sensitize nociceptor neurons involved in other pain modalities such as the mechano-sensation, as is the case of a non-peptidergic neuronal population characterized by the binding of isolectin B4 and expression of the Mrg family of G-protein-coupled receptors subfamily D [28,57]. However, as discussed above, a possible neuro-immune interaction was insufficient to alter Ehrlich tumor progression in the context of TRPV1 deficiency.

Our study aimed at investigating the contribution of the primary afferent nociceptive neurons expressing TRPV1 in the nociceptive behaviors triggered upon Ehrlich tumor administration in mice. We considered that the primary afferent neurons are in contact with the tumor cells and make this communication between the peripheral tissues and the spinal cord, making them an important group of cells. However, it is important to mention other possibilities for future studies. As far as we know, there is no study investigating central sensitization in the Ehrlich tumor model or whether TRPV1^+^ primary afferent neurons or afferent terminals of TRPV1^+^ neurons in the spinal cord contribute to central sensitization in this model. For instance, in the spinal cord dorsal horn, TRPV1^+^ glutamatergic neurons functionally contribute to excitatory neurotransmission [58]. Spinal NMDA (N-methyl-D-aspartate) is important in cancer pain. Disrupting the neuronal transport of NR2B (NMDA receptor containing a 2B subunit) in the spinal cord reduces the bone pain caused by injecting fibrosarcoma NCTC 2472 cells into the femur [59]. Evidence also supports that glutamatergic signaling via NR2B activates neuronal PKCγ (protein kinase C gamma) as a mechanism to induce central sensitization in cancer bone pain [60]. Thus, it is also possible that spinal TRPV1^+^ neuronal afferent terminals contribute to central sensitization in Ehrlich tumor pain via glutamate, although this hypothesis remains to be investigated.

## 5. Conclusions

In conclusion, we demonstrate that TRPV1 is involved in Ehrlich tumor pain-like behavior without interference in tumor progression. This mechanism was unknown for this model, thus, further validating Ehrlich tumor as a pre-clinical in vivo model for investigating disease mechanisms and drug development in cancer.

## Data Availability

The data presented in this study are available on reasonable request to the corresponding author.

## Supplementary Materials

The following supporting information can be downloaded at: https://www.mdpi.com/article/10.3390/brainsci12091247/s1, Table S1: Statistical information from results shown in Figure 1; Table S2: Statistical information from results shown in Figure 2; Table S3: Statistical information from results shown in Figure 3.
