# Supplementary material for: Ehrlich Tumor Induces TRPV1-Dependent Evoked and Non-Evoked Pain-like Behavior in Mice
Source: Brain Sci. 2022 Sep 15;12(9):1247. doi: 10.3390/brainsci12091247 (PMC9496717; doi:10.3390/brainsci12091247)
Supplement: Supplementary file 1 [file brainsci-12-01247-s001.zip › Table S1 MMB BRAIN SCIENCES .pdf]

Table S1. Statistical information from results shown in Figure 1.

Result				Shapiro-Wilk test		Brown-Forsythe		Statistical test			
	**Group**	**N Animals per plate**	**N experimental**	**W value**	**P value**	**F value**	**P value**		**Kruskal-Wallis statistic**	**Post-Test**	**P value**
**Figure 1E**	WT+ Saline Baseline	10	2	0,8475	<0,0001	25,43 (3, 362)	<0,0001	Kruskal-Wallis	338,6	Dunn	<0,0001
	WT + Ehrlich Baseline	10	2	0,9361	0,0002						
	WT+ Saline Caps	10	2	0,7654	<0,0001						
	WT + Ehrlich Caps	10	2	0,8072	<0,0001						
