# Supplementary material for: Ehrlich Tumor Induces TRPV1-Dependent Evoked and Non-Evoked Pain-like Behavior in Mice
Source: Brain Sci. 2022 Sep 15;12(9):1247. doi: 10.3390/brainsci12091247 (PMC9496717; doi:10.3390/brainsci12091247)
Supplement: Supplementary file 1 [file brainsci-12-01247-s001.zip › Table S2 MMB BRAIN SCIENCES .pdf]

Table S2. Statistical information from results shown in Figure 2.

Result			Shapiro-Wilk test		Brown-Forsythe			Statistical tests			
	**Group**	**n**	**W value**	**P value**	**Time points**	**F value F (DFn, DFd)**	**P value**	**Test**	**Post-Test**	**F value**	**P value**
**Figure 2B**	WT + Saline	10	0,9049	0,3615	Baseline	0,07920 (2, 27)	0,9241	Two-way ANOVA	Tukey	Interaction F (12, 162) = 55,36	P<0,0001
					2 days	1,546 (2, 27)	0,2315			Row Factor / Time F (4,901, 132,3) = 67,39	P<0,0001
	WT + Ehrlich	10	0,9253	0,5114	4 days	3,030 (2, 27)	0,0650				
					6 days	0,6284 (2, 27)	0,5411				
					8 days	1,745 (2, 27)	0,1938			Colum Factor/ Group F (2, 27) = 302,4	P<0,0001
					10 days	2,886 (2, 27)	0,0732				
	TRPV1 -/- + Ehrlich	10	0,8828	0,2392	12 days	3,346 (2, 27)	0,0503			Subject F (27, 162) = 3,314	P<0,0001
**Figure 2C**	WT + Saline	10	0,9848	0,9796	Baseline	2,804 (2, 27)	0,0783	Two-way ANOVA	Tukey	Interaction F (12, 162) = 18,58	P<0,0001
					2 days	1,083 (2, 27)	0,3527			Row Factor / Time F (3,919, 105,8) = 18,92	P<0,0001
	WT + Ehrlich	10	0,8250	0,0717	4 days	2,042 (2, 27)	0,1494				
					6 days	2,388 (2, 27)	0,1109			Colum Factor/ Group F (2, 27) = 211,0	P<0,0001
	TRPV1 -/- + Ehrlich	10	0,9450	0,6841	8 days	2,779 (2, 27)	0,0799				
					10 days	3,102 (2, 27)	0,0613			Subject F (27, 162) = 2,557	P=0,0002
					12 days	2,288 (2, 27)	0,1208				

**Figure 2D**	WT + Saline	10	0,9671	0,8766	Baseline	1,373 (2, 27)	0,2705	Two-way ANOVA	Tukey	Interaction F (12, 162) = 80,06	P<0,0001
					2 days	1,978 (2, 27)	0,1579				
	WT + Ehrlich	10	0,9219	0,4842	4 days	2,692 (2, 27)	0,0859			Row Factor / Time F (2,236, 60,38) = 304,0	P<0,0001
					6 days	2,717 (2, 27)	0,0841				
					8 days	2,472 (2, 27)	0,1033			Colum Factor/ Group F (2, 27) = 162,7	P<0,0001
	TRPV1 -/- + Ehrlich	10	0,9218	0,4837	10 days	2,079 (2, 27)	0,1446			Subject F (27, 162) = 5,877	P<0,0001
					12 days	3,218 (2, 27)	0,0558				
**Figure 2E**	WT + Saline	10	0,9445	0,6041	n.a.	10,59 (2, 27)	0,0004	Kruskal-Wallis	Dunn	19,38	P<0,0001
	WT + Ehrlich	10	0,9284	0,4327							
	TRPV1 -/- + Ehrlich	10	0,9245	0,3957							
**Figure 2F**	WT + Saline	10	0,9497	0,7265	Baseline	0,05123 (2, 27)	0,9502	Two-way ANOVA	Tukey	Interaction F (12,162) = 12,89	P<0,0001
					2 days	0,3113 (2, 27)	0,7351			Row Factor / Time F (4,498, 121,4) = 36,29	P<0,0001
	WT + Ehrlich	10	0,9148	0,4298	4 days	0,1421 (2, 27)	0,8682				
					6 days	2,782 (2, 27)	0,0797			Colum Factor/ Group F (2, 27) = 167,7	P<0,0001
					8 days	3,213 (2, 27)	0,0560				
	TRPV1 -/- + Ehrlich	10	0,9200	0,4694	10 days	1,478 (2, 27)	0,2459				
					12 days	1,061 (2, 27)	0,3600			Subject F (27, 162) = 1,048	P=0,4088
