# Supplementary material for: Ehrlich Tumor Induces TRPV1-Dependent Evoked and Non-Evoked Pain-like Behavior in Mice
Source: Brain Sci. 2022 Sep 15;12(9):1247. doi: 10.3390/brainsci12091247 (PMC9496717; doi:10.3390/brainsci12091247)
Supplement: Supplementary file 1 [file brainsci-12-01247-s001.zip › Table S3 MMB BRAIN SCIENCES .pdf]

Table S3. Statistical information from results shown in Figure 3

Result			Shapiro-Wilk test		Brown-Forsythe			Statistical tests			
	Group	n	W value	P value	Time points	F value F (DFn, DFd)	P value	Test	Post-Test	F value	P value
Figure 3B	WT + Saline	8	0,8688	0,2617	Baseline	0,4078 (2, 21)	0,6703	Two-way ANOVA	Tukey	Interaction F (8, 84) = 45,37	P<0,0001
					0h	2,175 (2, 21)	0,1385			Row Factor / Time F(3,389, 71,17) = 43,21	P<0,0001
	WT + Ehrlich + vehicle	8	0,8586	0,2231	1h	0,4126 (2, 21)	0,6672				
					3h	0,5027 (2, 21)	0,6120			Colum Factor/ Group F (2, 21) = 989,2	P<0,0001
	WT + Ehrlich + AMG9810	8	0,9193	0,5255	5h	2,651 (2, 21)	0,0941				
					7h	1,042 (2, 21)	0,3704			Subject F (21, 84) = 1,495	P=0,4194
Figure 3C	WT + Saline	10	0,9848	0,9796	Baseline	3,384 (2, 21)	0,0532	Two-way ANOVA	Tukey	Interaction F (8, 84) = 11,25	P<0,0001
					0h	0,2971 (2, 21)	0,7460			Row Factor / Time F (2,583, 54,24) = 8,543	P=0,0002
	WT + Ehrlich + vehicle	10	0,8250	0,0717	1h	3,149 (2, 21)	0,0637				
					3h	1,954 (2, 21)	0,1666			Colum Factor/ Group F (2, 21) = 194,7	P<0,0001
	TRPV1 -/- + Ehrlich	10	0,9450	0,6841	5h	1,530 (2, 21)	0,2398				
					7h	0,4864 (2, 21)	0,6216			Subject F (21, 84) = 1,971	P=0,0158
